# Indirubin attenuates mouse psoriasis-like skin lesion in a CD274-dependent manner: an achievement of RNA sequencing

**DOI:** 10.1042/BSR20180958

**Published:** 2018-11-23

**Authors:** Xiaochun Xue, Jianhua Wu, Junhui Li, Jianguo Xu, Haiying Dai, Congshan Tao, Chao Li, Jinhong Hu

**Affiliations:** 1 Department of Pharmacy, Changhai Hospital, Second Military Medical University, Shanghai 200433, China; 2 Department of Pharmacy, the 85th Hospital of PLA, Shanghai 200052, China; 3 Department of Dermatology, Changhai Hospital, Second Military Medical University, Shanghai 200433, China; 4 Department of Plastic Surgery, Changhai Hospital, Second Military Medical University, Shanghai 200433, China; 5 Department of Pharmacy, Wuxi Maternal and Child Health-Care Hospital, Wuxi 214000, China; 6 Department of Pharmacy, Xinhua Hospital, School of Medicine, Shanghai Jiao Tong University, Shanghai 200092, China

**Keywords:** differentially expressed genes, indirubin, IFN-γ, psoriasis, PD-L1, RNA sequencing

## Abstract

It was previously reported that the expression of CD274 was down-regulated in psoriatic epidermis, leading to immune disorders of psoriasis. However, the regulatory mechanisms of CD274 were rarely elucidated. We aimed to explore the regulatory mechanisms of CD274. Skin samples were collected from 18 patients with psoriasis vulgaris and 9 healthy participants for RNA sequencing. Candidate genes were chosen based on degree and k-core difference of genes in the co-expression network. The relations between candidate genes and CD274 were validated by flow cytometry and real-time PCR in primary human epidermal keratinocytes. The therapeutic effect of indirubin was assessed in an imiquimod-treated mouse model. Interferon-γ (IFN-γ), cyclin-dependent kinase (CDK) 1, Toll-like receptor 3 (TLR3), TLR4 and interleukin (IL)-17A were considered as candidate genes. In primary human epidermal keratinocytes, the level of CD274 was obviously increased under the stimulation of IFN-γ and CDK1 inhibitor (indirubin), independent of TLR4, TLR3 or IL-17A. Indirubin alleviated the severity of psoriatic mice in a CD274-dependent manner. Co-expression network analysis served as an effective method for the exploration of molecular mechanisms. We demonstrated for the first time that CD274 was the regulator of indirubin-mediated effect on mouse psoriasis-like skin lesion based on co-expression network analysis, contributing to the alleviation of mouse psoriasis-like skin lesion.

## Introduction

Psoriasis vulgaris is one of the most common forms of psoriasis, which is related to T-lymphocyte infiltration in skin [1]. CD274, also known as programmed cell death ligand 1 or B7-H1, was reported to inhibit T-cell differentiation and proliferation thus playing a vital role in the regulation of immune reaction [2,3]. It has been proven that the level of CD274 is decreased in the psoriatic epidermis compared with normal epidermis. Decreased CD274 protein was considered to involve the immune disorders of psoriasis [4]. Furthermore, in the imiquimod (IMQ)-induced psoriatic mouse models, the administration of recombinant CD274 protein decreased psoriatic inflammation and alleviated psoriatic symptoms [5]. Tumor patients were reported to induce psoriasis under the treatment of CD274 antibody [6–8]. These previous studies indicated the vital role of CD274 in psoriasis. Therefore, drugs increasing the level of CD274 in keratinocytes may serve as a potential therapeutic strategy in the treatment of psoriasis. However, the regulatory mechanisms of CD274 in keratinocytes are not fully elucidated and drugs of regulating CD274 have not been found.

In the past 10 years, RNA sequencing has been developed as a popular deep-sequencing technology. Compared with other transcriptomics methods, it enables the much more rapid and cost-effective generation of massive amounts of sequences and provides a more precise measurement of levels of transcripts [9]. The technology of RNA sequencing is an effective gene expression profiling method, which has been widely applied in various fields including biological medicine. According to the bioinformatics analysis of differential genes, biomarkers and drug targets have been discovered for the diagnosis and treatment of diseases. Besides, the pathological mechanisms of disease and functions of gene can be evaluated [10–12]. In the field of psoriasis, RNA sequencing has been used to explore many functions of genes, such as defensin-like antimicrobial activity of *LCE* 3 [13] and transcription factor FOXA1 to inhibit the formation of regulatory T-cells subpopulation [14].

In the present study, we conducted RNA sequencing in skin tissues from psoriatic and healthy persons to discuss the CD274-dependent regulatory mechanisms. Cellular and animal experiments were conducted for further detection to ultimately develop CD274-dependent regulatory drugs. We raised a hypothesis that CD274 was the regulator of indirubin-mediated effect on mouse psoriasis-like skin lesion, which contributed to the alleviation of mouse psoriasis-like skin lesion. We believe that the present study might provide a novel insight in the exploration of mechanisms in the pathogenesis of psoriasis vulgaris and development of drugs.

## Materials and methods

### Participants and samples

Normal skins for control were obtained from nine volunteers undergoing surgical operations in the Department of Plastic Surgery, Changhai Hospital, Shanghai, China. Volunteers were observed neither systemic or autoimmune diseases nor relevant family history. Psoriatic skins were obtained from 18 patients with psoriasis vulgaris in the same institution. No patients had received systemic or topical therapies for 4 weeks before the skins were obtained. Ethical approval was obtained from the Ethics Committee of Changhai Hospital and written informed consent was signed by all of the participants in the present study. All of the samples were immediately snap-frozen in liquid nitrogen and stored at −80°C for RNA extraction.

### RNA sequencing

Whole skin from psoriatic patients and healthy people was used for total RNA extraction by TRIzol Reagent according to the manufacturer’s protocol (Thermo Scientific, Bremen, Germany). RNA concentration and quality were detected by Agilent 2200. Only RNA samples with RNA integrity number more than 6.0 were used to construct a complementary DNA library. The complementary DNA library was prepared using an Ion Total RNA-Seq Kit v2 (Thermo Scientific, Bremen, Germany) according to the manufacturer’s protocol. The quantity and quality of obtained complementary DNA libraries were assessed by an Agilent Bioanalyzer 2200 system (Agilent Technologies, Inc., Santa Clara, CA, U.S.A.). The complementary DNA libraries were then processed for protein sequencing according to commercially available protocol. The sequencing quality was analyzed using RSeQC [15]. Principal component analysis was applied to differentiate if there was a significant distinction between two groups [16].

### Bioinformatics analysis

Differentially expressed genes (DEGs) between psoriatic skins and normal skins were filtered by means of DE-seq. The values of fold change (FC) and statistical significance (*P*) were calculated. False discovery rate (FDR) was detected to correct the *P* value [17]. After the assessment of FDR and FC, we selected the DEGs with FDR < 0.05 and |log_2_FC| > 1 [18]. The function and pathway of the DEGs were analyzed according to the Gene Ontology (GO) database [19,20] and KEGG database, respectively [21]. Fisher’s exact test and χ^2^ test were applied to classify the GO category and select the significant pathway. According to the significant difference from both GO and pathway (*P*<0.01), we selected the relevant DEGs and 30 long non-coding RNA with high values of |log_2_FC| to build gene co-expression networks. For each pair of genes, we calculated the Pearson Correlation and chose the significant correlation pairs (FDR < 0.01) to construct the network [22]. In the network, the importance of genes was determined by the degree (the line numbers that one gene links to the other) and the k-core (genes that are connected to at least k other genes) [23–26].

### Cell culture and treatment

Primary human epidermal keratinocytes served as the *in vitro* model for the present study. Epidermal keratinocytes were obtained from adult foreskin isolated from patients in the Department of Plastic Surgery, Shanghai Changhai Hospital. Foreskin was rinsed in EpiLife^™^ medium with 20 μg/ml gentamycin solution and cut into strips (0.5 cm × 1 cm) after removing subcutaneous mucosa. The cut pieces were submerged in the 0.2% dispase II (neutral protease, grade II) solution (Roche, Indianapolis, IN, U.S.A.) and then incubated in a 4°C refrigerator for 16 h. Epidermis was separated from the dermis and incubated in the recombinant trypsin/EDTA solution for 30 min at a 37°C water bath. After neutralized by the defined trypsin inhibitor, the solution was filtered in a 40 μm cell strainer. Keratinocytes were resuspended in EpiLife^™^ medium with the addition of human keratinocyte growth supplement (Gibco, Carlsbad, CA, U.S.A.) after centrifugation and were seeded in a 25 cm^2^ flask at 5 × 10^3^ cells/cm^2^. Cells at the passage of 2–3 were used for the experiments. After reaching semi-confluence, cells were stimulated with 25 ng/ml interferon-γ (IFN-γ; PeproTech, Rocky Hill, NJ, U.S.A.), 0.3 μg/ml polyinosinic-polycytidylic acid (poly (I: C); InvivoGen, Toulouse, France), 100 ng/ml IL-17A (PeproTech, Rocky Hill, NJ, U.S.A.), 20 μg/ml indirubin (National Institutes for Food and Drug Control, Beijing, China), 10 μg/ml lipopolysacchride (LPS; Sigma-Aldrich, St. Louis, MO, U.S.A.) as well as relative vehicle reagent (medium or DMSO) for 24 h, respectively. Finally, cells were harvested for flow cytometry or real-time PCR.

### Flow cytometry

Cells were washed three times with cold PBS and surface staining was performed at room temperature for 30 min using anti-human CD274 antibodies or mouse IgG1 kappa isotype control (eBioscience, San Diego, CA, U.S.A.). The results were analyzed by FlowJo software (Tree Star Inc., Ashland, OR, U.S.A.). The levels of CD274 were shown with mean fluorescence intensity (MFI).

### Real-time PCR

Total RNA of cells was extracted by TRIzol Reagent according to the manufacturer’s protocol. The concentration and purity of RNA were determined by NanoDrop 1000 spectrophotometer (Thermo Fisher Scientific, Wilmington, DE, U.S.A.). cDNA was synthesized according to the manufacturer’s protocol of PrimeScript™ RT Master Mix (Takara, Dalian, China). Levels of CD274 were assessed using TB Green™ Premix Ex Taq™ II (Takara, Dalian, China) and primers on LightCycler 480 II real-time PCR system (Roche, Mannheim, Germany). The thermal cycles was set at 95°C for 30 s, followed by 40 cycles of 95°C (10 s), 60°C (10 s) and 72°C (10 s). The expression of CD274 was normalized to the internal control by using comparative cycle threshold (*C*_T_) method where fold difference = 2^−ΔΔ*C*_T_^. GAPDH genes were used as an internal control. GAPDH forward primer: 5′-CAGGAGGCATTGCTGATGAT-3′ and reverse primer: 5′-GAAGGCTGGGGCTCATTT-3′. CD274 forward primer: 5′-GGTGCCGACTACAAGCGAAT-3′ and reverse primer: 5′-GGTGACTGGATCCACAACCAA-3′.

### Animals

Male BALB/c mice (8 weeks old) were obtained from the experimental animal center of the Second Military Medical University and bred in a specific pathogen-free condition. Mice were maintained on a 12-h light/dark cycle and supplied with food and water *ad libitum*. The animal protocols used in the present study were approved by the experimental animal ethics committee of the Second Military Medical University.

### IMQ-induced psoriasis-like mouse model and *in vivo* treatment

Mice were randomly classified into five groups, including the control, animal model, indirubin, indirubin together with anti-CD274 and CD274-Fc groups. Mice in the model group were treated with 5% IMQ cream (62.5 mg; 3M Health Care Limited, Loughborough, Leicestershire, U.K.) on the shaved dorsal skin for six consecutive days to generate psoriasis-like lesions, while mice in the control group were given vehicle cream. Indirubin (50 mg/kg) or mGoInVivo^™^ purified anti-mouse CD274 antibody (100 μg; Biolegend, San Diego, CA, U.S.A.) was subcutaneously given every day after IMQ treatment. Recombinant mouse CD274-Fc chimera protein (20 μg; R&D Systems, Minneapolis, MN, U.S.A.) was administrated intradermally onto the back every 12 h after IMQ treatment. Mice were killed on day 7 after the first day of IMQ application.

### Histological analysis

The dorsal skin of mice was fixed overnight in 4% paraformaldehyde solution and embedded in paraffin. The paraffin-embedded specimens were processed for hematoxylin and eosin staining according to the standard procedures.

### Immunofluorescence analysis

Frozen sections of mouse skin tissues were stained overnight with primary antibodies including rabbit anti-mouse cytokeratin (CK)-14 antibody (Abcam, Cambridge, MA, U.S.A.) and rat anti-mouse CD274 antibody (Abcam). After extensive washing, the sections were incubated with secondary antibodies including Alexa Fluo 594-conjugated donkey anti-rat IgG (Life Technologies, Carlsbad, CA, U.S.A.) and Alexa Fluor 488-conjugated donkey anti-rabbit IgG (Life Technologies) for 1 h at room temperature. After being washed, slides mounted with sections were stained for 2 min with 4′-6-diamidino-2-phenylindole (DAPI; Life Technologies) and the colocalization was detected by means of a fluorescence microscope (Olympus, Tokyo, Japan). The average number of fluorescent staining cells from mouse skin was counted from three independent images with a ×400 field.

### Statistical analysis

All data from *in vivo* and *in vitro* studies were expressed as means ± SD. Data were analyzed by SPSS Statistics 21 software (SPSS Inc., Chicago, IL, U.S.A.). *P* values were determined by student’s unpaired *t*-test or one-way ANOVA followed by Bonferroni’s test. *P* values of less than 0.05 were considered to be statistically significant.

### Ethics Statement

The present study was carried out in accordance with the recommendations of the biomedical research guidelines involving human participants established by the National Health and Family Planning Commission of China. Shanghai Changhai Hospital Ethics Committee approved the present study, and written informed consent was obtained from each subject in accordance with the Declaration of Helsinki.

## Results

### Quality of RNA sequencing and differential genes

**Figure 1:**
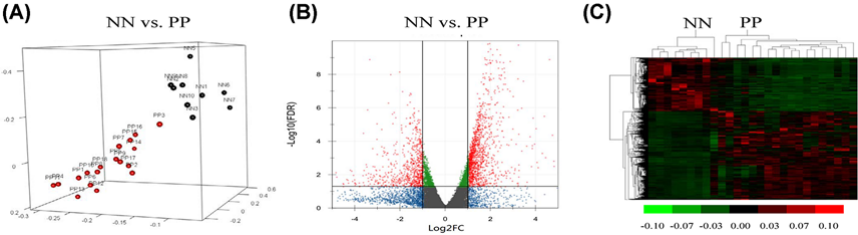
Genes between the skins from the PP and the NN were differentially expressed (**A**) Principal component analysis indicates the whole gene expression pattern of the two groups: PP (red balls) and NN (black balls). (**B**) The volcano plot represents all of the protein-coding genes in the two groups: genes with significantly differential expression (FDR <0.05, |log_2_FC| >1) (red dots) and genes without significantly differential expression (other color dots). (**C**) The heat map indicates the differentially expressed genes between the two groups. Each row or column represents one gene or sample, respectively. Green or red bar represents a decreased or increased gene expression, respectively. −0.1 represents the lowest expression value of the gene among all the samples and 0.1 represents the highest one.

The average age of psoriatic patients (PP) was 43.83 ± 16.29, which was 37.33 ± 13.73 in the normal controls (NN). No difference in gender or age was observed between the two groups. Detailed characteristics of the participants were listed in Table 1. The RNA quality of all samples conformed to the requirements of the sequencing. The raw sequence data yielded ∼2.3 gigabases of data per sample, with an average of 17 million reads (89.0% mapped rate) per sample. The average number of the GC content was ∼52% for each sample. Mapping of sequence reads yielded an average of 23,960 genes mapped for each sample. The figure of principal component analysis displayed a sharp separation between the two groups (Figure 1A). Finally, all samples were used to analyze the differential genes. The dataset analyzed during the present study was available in the NCBI Gene Expression Omnibus repository. The accession number for the dataset was GSE114286 (https://www.ncbi.nlm.nih.gov/geo/query/acc.cgi?acc=GSE114286).

**Table 1:** Characteristics of psoriatic patients and normal controls

	Psoriatic patients	Normal controls
	(*n*=18)	(*n*=9)
**Age (years)**		
Mean ± SD	43.83 ± 16.29	37.33 ± 13.73
Median	45.0 (14–68)	42.0 (20–55)
**Gender**		
Male/female	12/6	6/3
**PASI score, 0–72**		
Mean ± SD	21.61 ± 7.99	
Median	21.5 (8.6–41.3)	
**Psoriasis duration (years)**		
Mean ± SD	7.61 ± 8.75	
Median	4.0 (0–31)	
**Age of onset**		
Mean ± SD	36.28 ± 13.51	
Median	37.0 (13–58)	

Abbreviation: PASI, psoriasis area and severity index.

We then identified 2597 differential genes including protein-coding genes and un-coding RNA. Among all of the protein-coding genes, 1335 genes were up-regulated in the PP group, while 804 genes were down-regulated. Volcano plot and heat map were applied to describe the differential expression patterns of the protein-coding genes (Figure 1B,C).

### GO and pathway enrichment analysis

**Figure 2:**
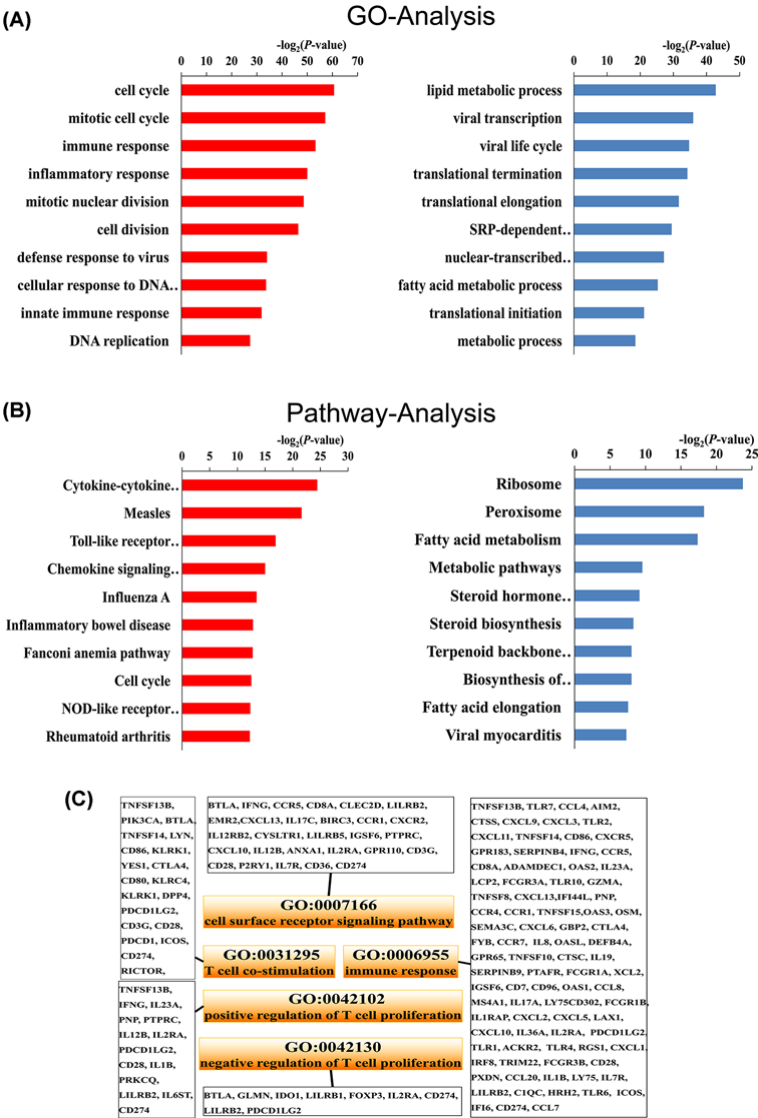
GO and pathway enrichment analysis of differentially expressed genes (**A**) The top 10 GO terms in the biological process clusters. (**B**) The top 10 pathways. The chart with red bars represents up-regulated and blue bars represent down-regulated genes (A and B). (**C**) The five GO terms related to CD274. Charts with orange bars represent the significantly enriched GO terms related to CD274 in the biological process and the corresponding genes are shown in the white boxes.

In order to learn more about the DEGs, GO analysis and pathway analysis were performed. According to the GO analysis, we found that the up-regulated genes were significantly enriched in 147 GO terms and there were 61 significantly down-regulated GO terms mapped to the GO database. According to the analysis of pathway enrichment, it was observed that there were 11 significantly down-regulated pathways and 33 up-regulated pathways. The top 10 GO terms and pathways were shown in Figure 2A,B, respectively. Additionally, GO terms related to CD274 were listed (Figure 2C). The functions of CD274 were related to the cell surface receptor signaling pathways, T-cell costimulation, immune responses, positive regulation of T-cell proliferation and negative regulation of T-cell proliferation. These results were consistent with the findings from other researches [27].

### Gene co-expression networks

**Figure 3:**
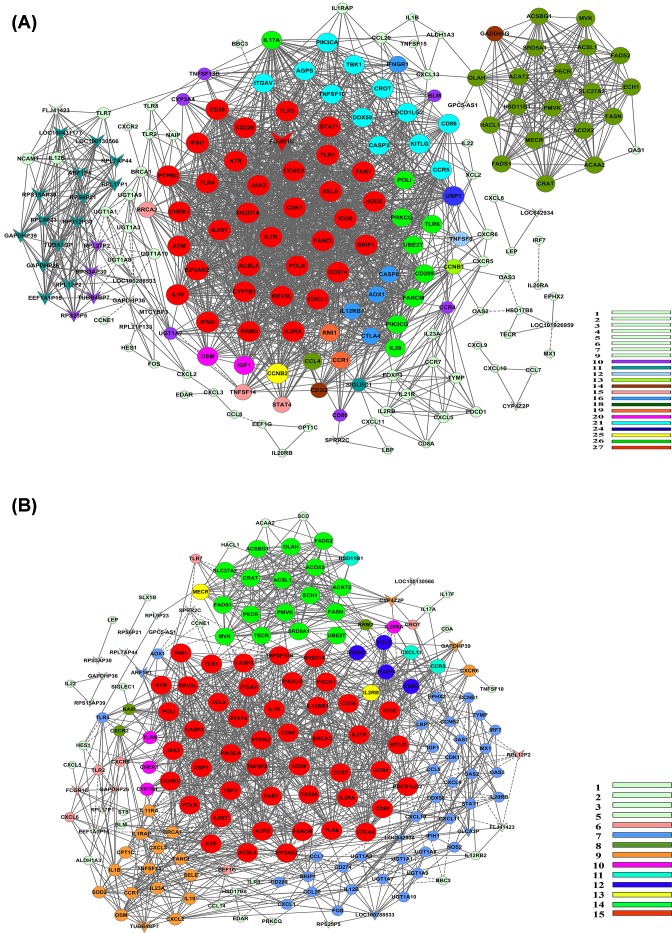
Gene co-expression networks of normal controls groups and psoriatic patients groups (**A** and **B**) Genes are described with nodes in the charts. The vee nodes represent non-coding RNA, and the circle nodes represent protein-coding genes. The k-core values of nodes in the networks are shown in different colors (the color bars are shown on the right bottom corner of each chart), and the volume of the nodes is in proportion to the values of k-core. The lines represent regulatory relations between two genes: positive correlations (solid lines) and negative correlations (long-dashed lines).

To fully predict the functions of genes, we constructed gene co-expression networks. The networks showed that there were markedly different co-expression patterns in the two groups (Figure 3). In the NN group, the co-expression network generated 188 nodes and 1972 connections. Among the 1972 pairs, 21 pairs presented as negative correlation, while the other pairs presented as positive correlation (Figure 3A). In the PP group, there were a total of 185 network nodes and 1349 connections among all of the genes. Among the 1349 pairs, 105 pairs presented as negative connections, while the other pairs presented as positive connections (Figure 3B). Genes with high k-core values were considered as key genes in the network, such as CD28, TLR3, TLR4, STAT1, JAK2, IFNG, CD274, CDK1, IL-17A and SOD2 in the NN group and JAK2, CD86, STAT4, CD28, IL12RB1, PDCD1, CD80 and PDCD1LG2 in the PP group. Genes related to CD274 in the networks were listed in Table 2. There were 56 genes with a positive correlation with CD274 in the NN group. Except one gene as negative, 16 genes showed a positive correlation with CD274 in the PP group. Based on the principle that genes with high values of differences in the degree (dif-degree) and k-core (dif-kcore) in the networks are considered as core genes in the disease [28], values of dif-degree and dif-kcore of genes listed in Table 2 were calculated and the top 20 genes were listed in Table 3. To detect whether there was connection between those genes and CD274, we further chose genes with values of dif-degree and dif-kcore higher than CD274 and verified in epidermal cells. Five candidate genes including IFN-γ, TLR4, CDK1, IL-17A and TLR3 were ultimately screened.

**Table 2:** Genes related to CD274 in the co-expression networks

Group	Gene	Pearson	*P*-value	FDR	Style
NN	IL2RA	0.9732955	9.98E-06	0.0001717	Positive
	PRKCQ	0.9687537	1.72E-05	0.0002295	Positive
	CD28	0.9636073	2.92E-05	0.0003181	Positive
	RRM2	0.9579704	4.81E-05	0.0004146	Positive
	CCR5	0.9491639	9.28E-05	0.0006202	Positive
	SH2D1A	0.9451843	0.0001203	0.0007168	Positive
	SOD2	0.9420219	0.000146	0.000804	Positive
	CDK1	0.9395096	0.0001689	0.0008682	Positive
	EIF2AK2	0.9374203	0.0001898	0.0009311	Positive
	STAT1	0.935628	0.0002092	0.0009775	Positive
	IL7R	0.9274773	0.0003149	0.0012596	Positive
	PTPRC	0.9266246	0.0003278	0.0012927	Positive
	CCNB2	0.9249148	0.0003547	0.0013297	Positive
	TLR4	0.9172249	0.0004951	0.0016521	Positive
	CD86	0.9127902	0.0005917	0.0018396	Positive
	SELE	0.90586	0.0007679	0.0021744	Positive
	CCR1	0.9043284	0.0008113	0.0022361	Positive
	ACSL4	0.8998301	0.0009485	0.0024332	Positive
	CHEK1	0.897294	0.0010326	0.0025454	Positive
	IFIH1	0.8901775	0.0012961	0.0029215	Positive
	IL6ST	0.8842571	0.0015483	0.0032586	Positive
	CASP8	0.8832669	0.0015936	0.003319	Positive
	TLR3	0.8818203	0.0016613	0.0033809	Positive
	IL19	0.8780748	0.001846	0.0036114	Positive
	FCGR1C	0.8780748	0.001846	0.0036114	Positive
	ATR	0.8765252	0.0019264	0.0036989	Positive
	JAK2	0.8721792	0.0021647	0.0039831	Positive
	FAR1	0.8709298	0.0022368	0.0040843	Positive
	CCNB1	0.8690707	0.0023472	0.0042117	Positive
	AOX1	0.8679496	0.0024155	0.0042772	Positive
	CTLA4	0.8556067	0.003261	0.0051653	Positive
	TLR6	0.854854	0.0033184	0.0052137	Positive
	IL12RB1	0.8535532	0.0034191	0.0052649	Positive
	PDCD1LG2	0.8534694	0.0034256	0.0052681	Positive
	ATM	0.8529131	0.0034694	0.0052953	Positive
	CD3G	0.8522091	0.0035253	0.0053394	Positive
	STAT4	0.8517455	0.0035625	0.0053668	Positive
	TNFSF14	0.8484067	0.0038381	0.0056231	Positive
	TLR1	0.8479869	0.0038737	0.0056293	Positive
	UBE2T	0.8459649	0.0040487	0.0058049	Positive
	POLK	0.8386417	0.0047275	0.0063276	Positive
	BRIP1	0.8381076	0.0047799	0.0063603	Positive
	IL26	0.8170625	0.0071733	0.0081403	Positive
	IL17A	0.8052876	0.0088147	0.0092264	Positive
	CD80	0.8052343	0.0088226	0.0092292	Positive
	TNFSF8	0.8038217	0.0090353	0.0093678	Positive
	CCL4	0.7989072	0.0098021	0.0098881	Positive
	CD209	0.9464572	0.000111	0.0006948	Positive
	CD226	0.8807744	0.0017115	0.0034349	Positive
	CXCL1	0.8978401	0.0010141	0.0025218	Positive
	CYP7B1	0.9328796	0.0002415	0.0010628	Positive
	FANCI	0.9250662	0.0003522	0.0013281	Positive
	ICOS	0.9539927	6.57E-05	0.0004822	Positive
	IFN-γ	0.8780748	0.001846	0.0036114	Positive
	PIK3CG	0.956161	5.56E-05	0.0004454	Positive
	REV3L	0.9490468	9.35E-05	0.0006202	Positive
PP	EEF1G	-0.675341	0.002101	0.0037489	Negative
	IL12B	0.901116	3.36E-07	9.64E-06	Positive
	CCL20	0.8556256	5.97E-06	7.67E-05	Positive
	CCL7	0.7927733	8.72E-05	0.0004859	Positive
	DDX58	0.7621884	0.0002362	0.0009399	Positive
	FOS	0.7618061	0.0002389	0.0009452	Positive
	IL19	0.664267	0.0026406	0.0043512	Positive
	IL1RAP	0.6642463	0.0026417	0.0043512	Positive
	IFIH1	0.6626158	0.00273	0.0044301	Positive
	STAT1	0.6597544	0.0028909	0.0045988	Positive
	CXCL10	0.6595464	0.0029029	0.0046016	Positive
	CXCL11	0.6376404	0.004416	0.0060023	Positive
	NOS2	0.628715	0.0051931	0.0066592	Positive
	CXCL1	0.6198472	0.006072	0.0072939	Positive
	CXCL2	0.6630916	0.002704	0.0044047	Positive
	LOC100288533	0.6543663	0.003215	0.0048891	Positive
	LOC642934	0.6395765	0.0042606	0.0058708	Positive

Abbreviations: FDR, false discovery rate; NN, normal controls; PP, psoriatic patients.

**Table 3:** Degree and k-core of the top 20 genes

GENE	Degree		K-core		Dif-Degree	Dif-Kcore
	Case	Control	Case	Control		
IFN-γ	0	41	0	27	-41	-27
IL26	0	38	0	26	-38	-26
TLR3	2	51	2	27	-49	-25
PRKCQ	2	37	2	26	-35	-24
IL17A	4	43	3	26	-39	-23
CDK1	10	53	7	27	-43	-20
TLR4	13	55	7	27	-42	-20
CD274	17	56	7	27	-39	-20
CXCL1	11	44	7	27	-33	-20
BRIP1	11	38	7	27	-27	-20
TNFSF10	1	26	1	21	-25	-20
CD209	9	45	7	26	-36	-19
RRM2	13	45	8	27	-32	-19
SOD2	14	55	9	27	-41	-18
FANCI	17	52	9	27	-35	-18
SELE	11	43	9	27	-32	-18
IL19	18	41	9	27	-23	-18
CCNB2	11	32	7	25	-21	-18
CYP7B1	12	57	10	27	-45	-17
CHEK1	11	52	10	27	-41	-17

Abbreviations: Dif-Degree, degree differences; Dif-Kcore, k-core differences.

### Increased expression of CD274 in primary human epidermal keratinocytes stimulated by IFN-γ and indirubin

**Figure 4:**
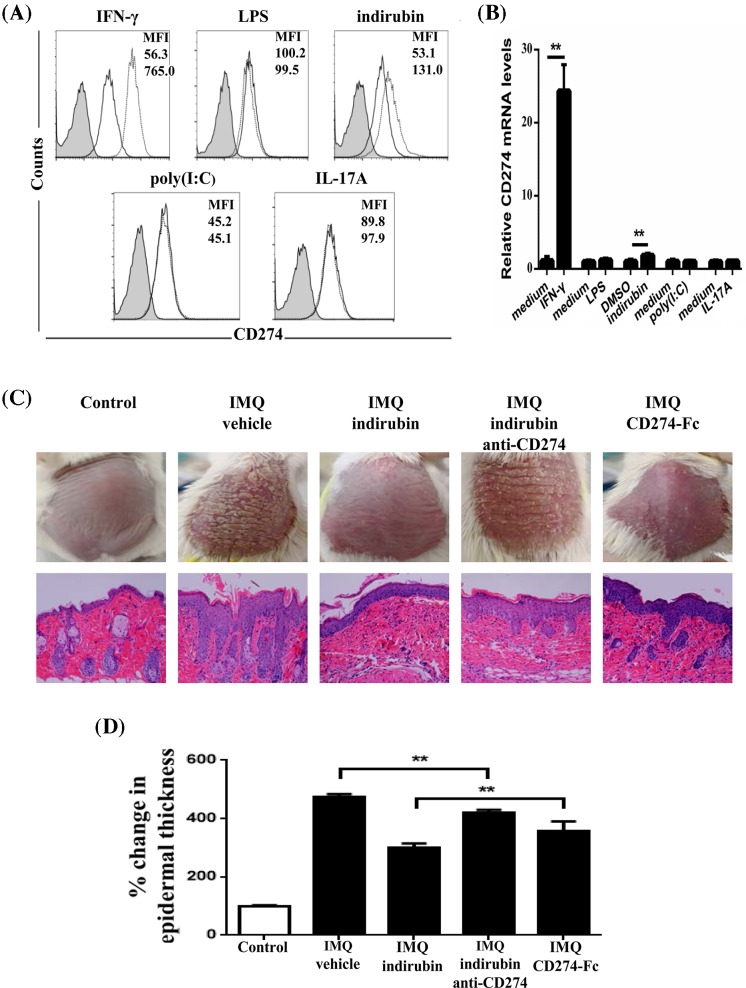
Expression of CD274 with several stimulations in primary human epidermal keratinocytes and *in vivo* effects of indirubin in an imiquimod (IMQ)-induced mouse model (**A** and **B**) Keratinocytes were stimulated with 25 ng/ml IFN-γ, 10 μg/ml LPS, 20 μg/ml indirubin, 0.3 μg/ml polyinosinic-polycytidylic acid (poly (I: C)), 100 ng/ml IL-17A as well as relative control reagent (medium or DMSO) for 24 h (*n*=6). (**A**) The expression of CD274 in protein was detected by flow cytometry using APC-conjugated antibodies. Representative profiles were shown as histograms, including isotype control antibody (shaded histogram with solid line) and CD274 antibody in the absence (solid line histogram) or presence (dotted line histogram) of stimulations. The levels of CD274 were shown in MFI) (B) The mRNA expression of CD274 was analyzed by real-time PCR. (**C** and **D**) Attenuation of psoriasis-like lesion on the back skins of IMQ-induced mice was observed by indirubin, CD274-Fc or anti-CD274 together with indirubin. Repeated IMQ treatment onto the dorsal skins for 6 days resulted in the generation of mouse psoriasis-like lesions. Indirubin, recombinant mouse CD274-Fc chimera protein and anti-mouse CD274 antibody were injected after IMQ treatment. Mice were killed on day 7 after the first day of IMQ application (*n*=6). (**C**) Representative photographs and images of hematoxylin and eosin staining of dorsal skin were shown, bar = 50 μm. (**D**) Epidermal thickness of the dorsal skin was expressed as the relative percentage over the measurement in untreated mice. Data were presented as mean ± SD. **P<0.01.

Since LPS (the activator of TLR4) and IFN-γ was previously reported to increase the expression of CD274 in oral keratinocytes [3,27], we then explored whether similar effects were detected in epidermal keratinocytes. We demonstrated that IFN-γ could significantly increase CD274 both in protein and mRNA levels in primary human epidermal keratinocytes. However, no such effect was observed under the challenge of LPS (Figure 4A,B). IL-17A, mainly secreted by TH17 and γδT cells, played an important role in psoriasis through acting on keratinocytes to induce a series of changes in epidermis [29]. Furthermore, poly (I: C), a TLR3 agonist, could attenuate the inflammatory reaction in psoriasis-like mouse models [30]. However, neither poly (I: C) nor IL-17A could change the expression of CD274 in epidermal keratinocytes (Figure 4A,B). Indirubin, a CDK1 inhibitor, was the main active ingredient of indigo naturalis, which belonged to a kind of traditional Chinese medicine. It has been demonstrated in clinical studies that external use of indigo naturalis could treat psoriasis mainly through its main active ingredient, indirubin. We further demonstrated that indirubin could increase the level of CD274 in epidermal keratinocytes (Figure 4A,B).

### Indirubin attenuates the symptoms of mice psoriasis in a CD274-dependent manner

Indirubin has been demonstrated to increase the expression of CD274 in human epidermal keratinocytes, we then detected whether CD274 was involved in the alleviative effect of indirubin on psoriasis. Indirubin significantly alleviated the symptom of imiquimod-induced mice through the observation of reduction of erythema, scaling and epidermal thickness (Figure 4C,D). However, CD274 antibody significantly attenuated the therapeutic effect, indicating the involvement of CD274 in the alleviative effect of indirubin. Furthermore, indirubin showed better therapeutic effect than CD274-Fc, indicating that indirubin was a multi-target agent, with CD274 as one of its effector molecules.

### CD274 expressions on keratinocytes in IMQ-induced mouse models

**Figure 5:**
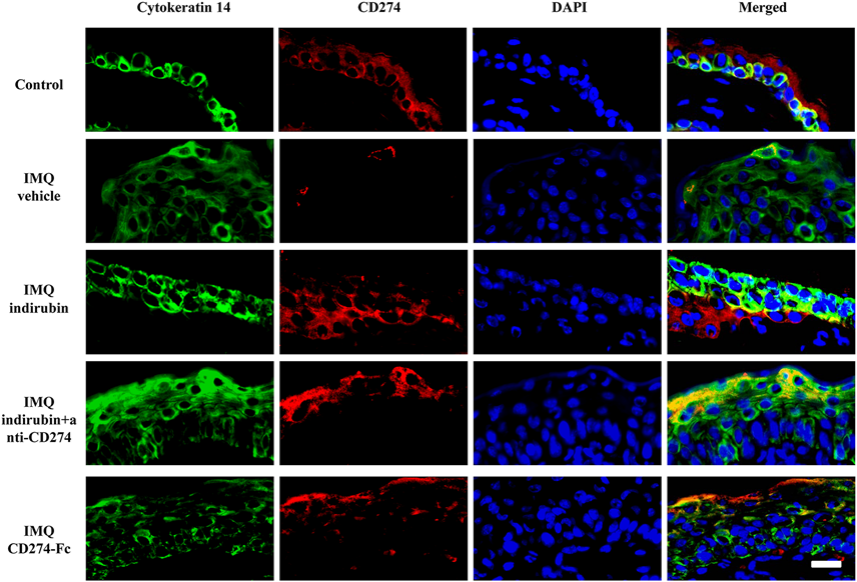
Expression of CD274 on keratinocytes in imiquimod (IMQ)-induced mouse models Mice were divided into five groups, including the control, IMQ, IMQ+indirubin, IMQ+indirubin+anti-CD274 and IMQ+CD274-Fc groups. Daily IMQ treatment onto the dorsal skins for 6 days resulted in the generation of mouse psoriasis-like lesions. For certain groups, indirubin, recombinant mouse CD274-Fc chimera protein or anti-mouse CD274 antibody was administrated after IMQ treatment. Mice were killed on day 7 after the first day of IMQ application. Frozen skin sections were costained with cytokeratin 14 antibody (to mark keratinocytes) and CD274 antibody by means of immunofluorescence. Representative images from the five groups are presented (scale bar = 20 μm).

We finally detected the expression of CD274 on keratinocytes in IMQ-induced mouse models. CK-14 antibody was used to identify skin keratinocytes (Figure 5). We found an extensive expression of CD274 in CK14^+^ keratinocytes of normal skin, while CD274 was seldom expressed in keratinocytes of IMQ group. After the treatment of indirubin, CD274 expression in keratinocytes was significantly increased in IMQ-induced mouse models, while those effects of indirubin were attenuated by the inhibition of CD274 antibody. Those data indicated the connection between indirubin and CD274 in keratinocytes in psoriasis (Figure 5).

## Discussion

Co-expression analysis has been applied to identify drug acting mechanisms and discover the biomarker of diseases [31,32]. In the present study, we initially performed RNA sequencing on skins from psoriatic patients and healthy persons to construct co-expression networks. By the analysis of 2139 DEGs, 208 significantly differential GO terms and 44 significantly differential pathways were generated. We discovered that the functions of DEGs were mainly related to cell cycle, inflammatory, virus, immune response and metabolic process. The major pathways were cytokine–cytokine receptor interaction, Toll-like receptor signaling pathway, chemokine signaling pathway, cell cycle, metabolic pathways, ribosome, peroxisome, steroid biosynthesis and biosynthesis of unsaturated fatty acids. These results were in line with the previously reported studies [10,32] and indicated that the pathogenesis of psoriasis was related to immunity, inflammation and metabolism. Finally, five candidate genes (IFN-γ, TLR4, CDK1, IL-17A and TLR3) related to CD274 were identified by the co-expression network analysis. Interestingly, the five genes were also reported to play important roles in psoriasis [10].

To our knowledge, this is the first application of co-expression network analysis to evaluate the mechanisms of CD274 in skin. Up-regulation of CD274 in immune cells and several cancer cells was reported to be mostly dependent on TLR or IFN-γ [33]. However, we discovered in the present study that it was IFN-γ but not TLR increased the expression of CD274 in human epidermal keratinocytes. In addition, CDK1 was demonstrated to serve as targets to increase the expression of CD274 by indirubin, an inhibitor of CDK1. Although the other three candidate genes failed to regulate the expression of CD274 in epidermal keratinocytes, yet the three genes were not without connection with CD274. Except for keratinocytes, there were other cells in skins, such as fibroblasts and immune cells. The three genes might produce correlations with CD274 in other skin cells. For instance, TLR3 agonist was reported to up-regulated the expression levels of CD274 on DCs [34]. Here, we mainly focus on the expression of CD274 in keratinocytes.

IFN-γ, mainly produced by type 1 T-helper cells in the skin, was demonstrated to be increased in expression in psoriatic lesions [35]. Similar increases were reported in the expression of IFN-γ-regulated genes [36]. IFN-γ could increase the secretion of inflammatory cytokines in keratinocytes, thus enhancing the inflammatory reaction in skin [37]. In addition, IFN-γ could modulate the immune reaction through the up-regulation of CD274 in endothelial cells, oral keratinocytes and dermal fibroblasts [27,38]. Consistent with those studies, we found in our current study that IFN-γ could increase the level of CD274, thus producing immunosuppressive effect. Those data provide the reason for the failure of IFN-γ antibody in the treatment of psoriasis despite of the pro-inflammatory effect of IFN-γ as shown previously [39].

CDK1 is associated with cell proliferation. In psoriatic skin lesion, the expression level of CDK1 is up-regulated, which is in line with the over proliferation of keratinocytes in the epidermis. Indirubin, an inhibitor of CDK1, has been reported to treat psoriasis effectively in several studies [40,41]. It suppressed the hyper-proliferation of epidermal keratinocytes, which was correlated with CDC25B gene expression [42]. On the other hand, indirubin inhibited EGF-induced EGFR phosphorylation and decreases the expressions of various inflammatory cytokines [42,43]. VEGFR-mediated JAK/STAT3 signaling was blocked by indirubin in endothelial cells [44]. In addition, indirubin could decrease the number of CD4^+^T cells and enhance the percentage of regulatory T cells in mice [45]. In our study, we also found the multi-target feature of indirubin, with the function related to CD274. Indirubin could increase the level of CD274 in epidermal keratinocytes and alleviate the symptom of psoriasis-like mice depending on CD274, indicating the value of indirubin in the treatment of psoriasis. The function of indirubin related to CD274 displayed its important role in immunoregulation.

In conclusion, in the present study, we have screened five candidate genes through RNA sequencing and found that IFN-γ and CDK1 were associated with CD274 through *in vitro* experiments. Further animal experiments demonstrated that CD274 could serve as one of the effector molecules of indirubin, a CDK1 inhibitor, against psoriasis. Although we have preliminarily uncovered the mechanisms of CD274 in epidermal keratinocytes through sequencing, the specific mechanisms in which indirubin and IFN-γ effect CD274 remain unclear. Studies are demanded for further exploration.

## Abbreviations

CDK: cyclin-dependent kinase
DEG: differentially expressed gene
EGFR: epidermal growth factor receptor
EGF: epidermal growth factor
FC: fold change
FDR: false discovery rate
IFN-γ: interferon-γ
IL: interleukin
IMQ: imiquimod
JAK/STAT3: janus kinase/signal transducers and activators of transcription 3
LPS: lipopolysacchride
MFI: mean fluorescence intensity
TLR: Toll-like receptor
VEGFR: vascular endothelial growth factor receptor

## References

[1] Boehncke W.-H. and Schön M.P. (2015) Psoriasis. Lancet North Am. Ed. 386, 983–994 10.1016/S0140-6736(14)61909-7 26025581

[2] Herold M., Posevitz V., Chudyka D. (2015) B7-H1 selectively controls TH17 differentiation and central nervous system autoimmunity via a novel non-PD-1-mediated pathway. J. Immunol. 195, 3584–3595 10.4049/jimmunol.1402746 26378076

[3] Zhang J., Tan Y.Q., Wei M.H. (2017) TLR4-induced B7-H1 on keratinocytes negatively regulates CD4+ T cells and CD8+ T cells responses in oral lichen planus. Exp. Dermatol. 26, 409–415 10.1111/exd.13244 27762043

[4] Kim D.S., Je J.H., Kim S.H. (2015) Programmed death-ligand 1, 2 expressions are decreased in the psoriatic epidermis. Arch. Dermatol. Res. 307, 531–538 10.1007/s00403-015-1588-5 26133691

[5] Kim J.H., Choi Y.J., Lee B.H. (2016) Programmed cell death ligand 1 alleviates psoriatic inflammation by suppressing IL-17A production from programmed cell death 1-high T cells. J. Allergy Clin. Immunol. 137, 1466e3–1476e3 26824999 10.1016/j.jaci.2015.11.021

[6] Troyanova-Slavkova S., Eickenscheidt L., Dumann K. and Kowalzick L. (2018) Initially undetected de novo psoriasis triggered by nivolumab for metastatic base of the tongue carcinoma. Hautarzt 69, 674–680 10.1007/s00105-017-4109-y 29330579

[7] Sibaud V. (2018) Dermatologic reactions to immune checkpoint inhibitors: skin toxicities and immunotherapy. Am. J. Clin. Dermatol. 19, 345–361 10.1007/s40257-017-0336-3 29256113

[8] Ruiz-Banobre J. and Garcia-Gonzalez J. (2017) Anti-PD-1/PD-L1-induced psoriasis from an oncological perspective. J. Eur. Acad. Dermatol. Venereol. 31, e407–e408 10.1111/jdv.14217 28295707

[9] Wang Z., Gerstein M. and Snyder M. (2009) RNA-Seq: a revolutionary tool for transcriptomics. Nat. Rev. Genet. 10, 57–63 10.1038/nrg2484 19015660 PMC2949280

[10] Jabbari A., Suarez-Farinas M., Dewell S. and Krueger J.G. (2012) Transcriptional profiling of psoriasis using RNA-seq reveals previously unidentified differentially expressed genes. J. Invest. Dermatol. 132, 246–249 10.1038/jid.2011.267 21850022 PMC3381505

[11] Li B., Tsoi L.C., Swindell W.R. (2014) Transcriptome analysis of psoriasis in a large case-control sample: RNA-seq provides insights into disease mechanisms. J. Invest. Dermatol. 134, 1828–1838 10.1038/jid.2014.28 24441097 PMC4057954

[12] Finotello F. and Trajanoski Z. (2018) Quantifying tumor-infiltrating immune cells from transcriptomics data. Cancer Immunol. Immunother. 67, 1031–1040 10.1007/s00262-018-2150-z 29541787 PMC6006237

[13] Niehues H., Tsoi L.C., van der Krieken D.A. (2017) Psoriasis-associated late cornified envelope (LCE) proteins have antibacterial activity. J. Invest. Dermatol. 137, 2380–2388 10.1016/j.jid.2017.06.003 28634035 PMC5651197

[14] Zolotarenko A., Chekalin E., Mehta R. (2017) Identification of transcriptional regulators of psoriasis from RNA-Seq experiments. Methods Mol. Biol. 1613, 355–370 10.1007/978-1-4939-7027-8_14 28849568

[15] Wang L., Wang S. and Li W. (2012) RSeQC: quality control of RNA-seq experiments. Bioinformatics 28, 2184–2185 10.1093/bioinformatics/bts356 22743226

[16] Zhao L., Yang S., Cheng Y. (2017) Identification of transcriptional biomarkers by RNA-sequencing for improved detection of beta2-agonists abuse in goat skeletal muscle. PLoS One 12, e0181695 10.1371/journal.pone.0181695 28746361 PMC5528896

[17] Pawitan Y., Michiels S., Koscielny S. (2005) False discovery rate, sensitivity and sample size for microarray studies. Bioinformatics 21, 3017–3024 10.1093/bioinformatics/bti448 15840707

[18] Anders S. and Huber W. (2010) Differential expression analysis for sequence count data. Genome Biol. 11, R106 10.1186/gb-2010-11-10-r106 20979621 PMC3218662

[19] Ashburner M., Ball C.A., Blake J.A. (2000) Gene ontology: tool for the unification of biology. The Gene Ontology Consortium. Nat. Genet. 25, 25–29 10.1038/75556 10802651 PMC3037419

[20] The Gene Ontology C. (2017) Expansion of the Gene Ontology knowledgebase and resources. Nucleic Acids Res. 45, D331–D338 10.1093/nar/gkw1108 27899567 PMC5210579

[21] Kanehisa M. and Goto S. (2000) KEGG: kyoto encyclopedia of genes and genomes. Nucleic Acids Res. 28, 27–30 10.1093/nar/28.1.27 10592173 PMC102409

[22] Prieto C., Risueno A., Fontanillo C. and De las Rivas J. (2008) Human gene coexpression landscape: confident network derived from tissue transcriptomic profiles. PLoS One 3, e3911 10.1371/journal.pone.0003911 19081792 PMC2597745

[23] Barabási A.-L. and Oltvai Z.N. (2004) Network biology: understanding the cell’s functional organization. Nat. Rev. Genet. 5, 101–113 10.1038/nrg1272 14735121

[24] Ravasz E., Somera A.L., Mongru D.A. (2002) Hierarchical organization of modularity in metabolic networks. Science 297, 1551–1555 10.1126/science.1073374 12202830

[25] Eidsaa M. and Almaas E. (2013) s-core network decomposition: a generalization of k-core analysis to weighted networks. Phys. Rev. E Stat. Nonlin. Soft Matter Phys. 88, 062819 10.1103/PhysRevE.88.062819 24483523

[26] Dorogovtsev S.N., Goltsev A.V. and Mendes J.F.F. (2006) k-core organization of complex networks. Phys. Rev. Lett. 96, 10.1103/PhysRevLett.96.040601 16486798

[27] Youngnak-Piboonratanakit P., Tsushima F., Otsuki N. (2004) The expression of B7-H1 on keratinocytes in chronic inflammatory mucocutaneous disease and its regulatory role. Immunol. Lett. 94, 215–222 10.1016/j.imlet.2004.05.007 15275969

[28] Ouyang Y., Guo J., Lin C. (2016) Transcriptomic analysis of the effects of Toll-like receptor 4 and its ligands on the gene expression network of hepatic stellate cells. Fibrogenesis Tissue Repair 9, 2 10.1186/s13069-016-0039-z 26900402 PMC4759739

[29] Burkett P.R. and Kuchroo V.K. (2016) IL-17 Blockade in Psoriasis. Cell 167, 1669 10.1016/j.cell.2016.11.044 27984714

[30] Cho K.A., Kim J.Y., Park M. (2017) Polyinosinic-polycytidylic acid (poly(I:C)) attenuates imiquimod-induced skin inflammation in mice by increasing cutaneous PD-L1 expression. Exp. Dermatol. 26, 346–348 10.1111/exd.13266 27897321

[31] Jia Z., Liu Y., Guan N. (2016) Cogena, a novel tool for co-expressed gene-set enrichment analysis, applied to drug repositioning and drug mode of action discovery. BMC Genomics 17, 414 10.1186/s12864-016-2737-8 27234029 PMC4884357

[32] Sundarrajan S. and Arumugam M. (2016) Weighted gene co-expression based biomarker discovery for psoriasis detection. Gene 593, 225–234 10.1016/j.gene.2016.08.021 27523473

[33] Ritprajak P. and Azuma M. (2015) Intrinsic and extrinsic control of expression of the immunoregulatory molecule PD-L1 in epithelial cells and squamous cell carcinoma. Oral. Oncol. 51, 221–228 10.1016/j.oraloncology.2014.11.014 25500094

[34] Pulko V., Liu X., Krco C.J. (2009) TLR3-stimulated dendritic cells up-regulate B7-H1 expression and influence the magnitude of CD8 T cell responses to tumor vaccination. J. Immunol. 183, 3634–3641 10.4049/jimmunol.0900974 19710456 PMC2789393

[35] Uyemura K., Yamamura M., Fivenson D.F. (1993) The cytokine network in lesional and lesion-free psoriatic skin is characterized by a T-helper type 1 cell-mediated response. J. Invest. Dermatol. 101, 701–705 10.1111/1523-1747.ep12371679 7693825

[36] Lew W., Bowcock A.M. and Krueger J.G. (2004) Psoriasis vulgaris: cutaneous lymphoid tissue supports T-cell activation and “Type 1” inflammatory gene expression. Trends Immunol. 25, 295–305 10.1016/j.it.2004.03.006 15145319

[37] Yang J.H., Yoo J.M., Lee E. (2018) Anti-inflammatory effects of Perillae Herba ethanolic extract against TNF-alpha/IFN-gamma-stimulated human keratinocyte HaCaT cells. J. Ethnopharmacol. 211, 217–223 10.1016/j.jep.2017.09.041 28970155

[38] Mazanet M.M. and Hughes C.C.W. (2002) B7-H1 is expressed by human endothelial cells and suppresses T cell cytokine synthesis. J. Immunol. 169, 3581–3588 10.4049/jimmunol.169.7.3581 12244148

[39] Harden J.L., Johnson-Huang L.M., Chamian M.F. (2015) Humanized anti-IFN-gamma (HuZAF) in the treatment of psoriasis. J. Allergy Clin. Immunol. 135, 553–556 10.1016/j.jaci.2014.05.046 25085340

[40] Lin Y.K., See L.C., Huang Y.H. (2018) Comparison of indirubin concentrations in indigo naturalis ointment for psoriasis treatment: a randomized, double-blind, dosage-controlled trial. Br. J. Dermatol. 178, 124–131 10.1111/bjd.15894 28815560

[41] Lin Y.K., See L.C., Huang Y.H. (2012) Comparison of refined and crude indigo naturalis ointment in treating psoriasis: randomized, observer-blind, controlled, intrapatient trial. Arch. Dermatol. 148, 397–400 10.1001/archdermatol.2011.1091 22431789

[42] Hsieh W.L., Lin Y.K., Tsai C.N. (2012) Indirubin, an acting component of indigo naturalis, inhibits EGFR activation and EGF-induced CDC25B gene expression in epidermal keratinocytes. J. Dermatol. Sci. 67, 140–146 10.1016/j.jdermsci.2012.05.008 22721997

[43] Kunikata T., Tatefuji T., Aga H. (2000) Indirubin inhibits inflammatory reactions in delayed-type hypersensitivity. Eur. J. Pharmacol. 410, 93–100 10.1016/S0014-2999(00)00879-7 11134660

[44] Zhang X., Song Y., Wu Y. (2011) Indirubin inhibits tumor growth by antitumor angiogenesis via blocking VEGFR2-mediated JAK/STAT3 signaling in endothelial cell. Int. J. Cancer 129, 2502–2511 10.1002/ijc.25909 21207415

[45] Zhang A., Qu Y., Zhang B. (2007) The different effects of indirubin on effector and CD4+CD25+ regulatory T cells in mice: potential implication for the treatment of autoimmune diseases. J. Mol. Med. 85, 1263–1270 10.1007/s00109-007-0235-9 17639287

